# Reduced Mu Power in Response to Unusual Actions Is Context-Dependent in 1-Year-Olds

**DOI:** 10.3389/fpsyg.2018.00036

**Published:** 2018-01-30

**Authors:** Miriam Langeloh, David Buttelmann, Daniel Matthes, Susanne Grassmann, Sabina Pauen, Stefanie Hoehl

**Affiliations:** ^1^Max Planck Institute for Human Cognitive and Brain Sciences, Leipzig, Germany; ^2^Department of Psychology, Heidelberg University, Heidelberg, Germany; ^3^Department of Psychology, University of Bern, Bern, Switzerland; ^4^Institute of Educational Research and Development, University of Applied Sciences and Arts Northwestern Switzerland, Windisch, Switzerland; ^5^Faculty of Psychology, University of Vienna, Vienna, Austria

**Keywords:** EEG, infants, action perception, action understanding, mu frequency, mirror neuron system

## Abstract

During social interactions infants predict and evaluate other people’s actions. Previous behavioral research found that infants’ imitation of others’ actions depends on these evaluations and is context-dependent: 1-year-olds predominantly imitated an unusual action (turning on a lamp with one’s forehead) when the model’s hands were free compared to when the model’s hands were occupied or restrained. In the present study, we adapted this behavioral paradigm to a neurophysiological study measuring infants’ brain activity while observing usual and unusual actions via electroencephalography. In particular, we measured differences in mu power (6 – 8 Hz) associated with motor activation. In a between-subjects design, 12- to 14-month-old infants watched videos of adult models demonstrating that their hands were either free or restrained. Subsequent test frames showed the models turning on a lamp or a soundbox by using their head or their hand. Results in the hands-free condition revealed that 12- to 14-month-olds displayed a reduction of mu power in frontal regions in response to unusual and thus unexpected actions (head touch) compared to usual and expected actions (hand touch). This may be explained by increased motor activation required for updating prior action predictions in response to unusual actions though alternative explanations in terms of general attention or cognitive control processes may also be considered. In the hands-restrained condition, responses in mu frequency band did not differ between action outcomes. This implies that unusual head-touch actions compared to hand-touch actions do not necessarily evoke a reduction of mu power. Thus, we conclude that reduction of mu frequency power is context-dependent during infants’ action perception. Our results are interpreted in terms of motor system activity measured via changes in mu frequency band as being one important neural mechanism involved in action prediction and evaluation from early on.

## Introduction

From birth on, infants take part in social interactions. These interactions with others are essential for the development of social-cognitive skills (Striano and Reid, 2006). An important ability trained in such interactions is to predict another person’s behavior and to react accordingly. This ability comprises that if the prediction turns out to be wrong (prediction error), the corresponding representation is updated appropriately (Kilner et al., 2007). Even though it is well established that the underlying action understanding starts developing early in life (Gredebäck and Daum, 2015), many open questions regarding its mechanisms remain. In the current study, we present evidence that motor activation in the mu frequency band is involved in infants’ action processing in the context of unknown objects and that infants take into account visible action constraints when evaluating actions on unknown objects.

Action understanding consists of both the ability to predict and to evaluate others’ actions (Gredebäck and Daum, 2015). The ability to *predict* what others will do next has been observed from 6 months on. By this age, infants show predictive eye movements to a target location of a goal-directed action involving everyday objects (e.g., phone or cup). In the second half of their 1st year, they predict more general action goals such as putting a ball into a bucket or bringing food or a cup to another person’s mouth (Falck-Ytter et al., 2006; Gredebäck and Melinder, 2010; Hunnius and Bekkering, 2010). The ability to *evaluate* actions has also been observed from 6 months on. Action evaluation is usually measured following the execution of an either expected or unexpected action (Gredebäck and Daum, 2015). Looking time studies demonstrate that infants look longer at actions with unexpected changes in the goal of a directional action (e.g., Woodward, 1998; Reid et al., 2007). Measuring pupil dilation in response to usual vs. unusual actions offers another method to gain insight into infants’ action evaluation. Pupil dilation usually follows after attention grabbing or unusual events (Libby et al., 1973). Gredebäck and Melinder (2010) found that 6- and 12-month-old infants’ pupils dilated in response to unusual feeding actions (e.g., spoon with food put to the hand). Hence, we already know that infants predict and evaluate another person’s behavior indicating a quite elaborate action understanding that emerges during the 1st year of life. Behavioral imitation studies provide yet another approach to examine infants’ action understanding, but are often used with slightly older children (e.g., Gampe et al., 2016). Interestingly, behavioral studies show that infants do not imitate every action they observe. They do so selectively depending on characteristics of the model, such as his or her reliability (Zmyj et al., 2010), group membership (Buttelmann et al., 2013) or external factors such as situational constraints (Gergely et al., 2002).

Gergely et al. (2002) investigated how infants imitate another person’s action according to efficiency and situational constraints. The authors found that 14-month-old infants were more likely to imitate an unusual head-touch action (i.e., turning on a lamp using the head) when the model’s hands were free compared to when her hands were occupied by holding a blanket. Gergely et al. (2002) concluded that this is because infants evaluated actions according to their efficiency or rationality in the given situation (Gergely and Csibra, 2003). This finding was replicated using similar paradigms and designs, and by testing even younger age groups (Schwier et al., 2006; Buttelmann et al., 2008; Zmyj et al., 2009; Gellén and Buttelmann, 2017). In particular, Zmyj et al. (2009) showed that 12- but not 9-month-old infants considered non-voluntary physical restraints (i.e., model’s hands tied to the table) when imitating unusual head-touch actions. However, divergent interpretations relating infants’ selective imitation behavior to more basic attention processes or motor resonance (i.e., to map others’ actions onto one’s own motor repertoire) have been brought forward (Paulus et al., 2011a,b; Beisert et al., 2012; but see also Buttelmann and Zmyj, 2012; Buttelmann et al., 2017).

Thus, in the present study, we measured infants’ neural responses when observing head-touch actions similar to the original paradigm by Gergely et al. (2002) in order to investigate possible neural mechanisms, particularly the role of motor activation during the observation of unusual actions. In contrast to previous imitation studies, we did not focus on behavioral responses (i.e., imitation rates) as dependent variable, but rather explored the role of motor activation in infants’ brains. The rationale of this approach is that selective motor activation during action observation is likely to be involved in action understanding, as action understanding is shaped by action skills. In particular, Hunnius and Bekkering (2014) found that any progress in motor development is typically associated with improved action understanding, resulting mainly from actively experiencing motor actions (see also Sommerville et al., 2005). This is in accordance with results that suggest that 10-month-olds’ motor actions develop ahead of their ability to predict action outcomes (Rosander and von Hofsten, 2011). In addition, Stapel et al. (2016) showed that infants who were experienced crawlers but not yet walkers were more accurate in predicting crawling actions than walking actions in an eye-tracking experiment (see also the eye-tracking study by Bache et al., 2017).

These studies suggest that one of the functional mechanisms underlying action understanding is the mirror neuron system (MNS). Mirror neurons discharge during both action observation and action execution (Rizzolatti et al., 2001; Rizzolatti and Craighero, 2004). Thus, observed actions seem to activate motor processes or schemas in the observer’s brain that would also be activated if the person executed the action themself (Prinz, 1997). Consequently, this motor system might be highly relevant for action prediction and evaluation (Wolpert and Flanagan, 2001; Prinz, 2006; Kilner et al., 2007).

One neural marker indicating motor activation and activation of the MNS during action observation and execution is the mu rhythm in the electroencephalogram (EEG) across central electrode sites. Mu rhythm activity has been examined in adults (e.g., Muthukumaraswamy et al., 2004; Lepage and Theoret, 2006) and in infants (e.g., van Elk et al., 2008; Southgate et al., 2009; Stapel et al., 2010; Marshall and Meltzoff, 2011; Cuevas et al., 2014). It is measured in the standard alpha frequency band (for adults at about 8–13 Hz and for infants at about 6–9 Hz) and is thought to reflect sensorimotor cortical activation (for a meta-analysis on EEG mu rhythm, see Pfurtscheller and Da Silva, 1999; Pineda, 2005; Fox et al., 2016). In particular, a suppression or desynchronization in the mu frequency band is associated with motor activation during action observation and execution. The decreasing mu power with movement onset indicates a decrease in neuronal synchrony reflecting the processing of movement-related information. Thus, mu rhythm is often interpreted as a neural correlate representing a link between action perception and production (Muthukumaraswamy et al., 2004).

Several infant studies suggest that the infant central mu rhythm is analogous to the adult mu rhythm (Marshall and Meltzoff, 2011). Southgate et al. (2009) demonstrated stronger mu desynchronization for observation and execution of reaching actions relative to baseline in 9-month-old infants. A second study showed similar results and reported stronger mu desynchronization in response to a reaching hand in a grasping posture even when the action outcome was not visible (Southgate et al., 2010). Thus, mu desynchronization additionally reflects infants’ prediction of the motor program of an anticipated action. Furthermore, significantly stronger mu desynchronization compared to baseline was found in 14-month-olds for the observation and execution of button presses in a live EEG paradigm (Marshall et al., 2011).

Mu desynchronization in infants seems to depend on active experience and, thus, on whether or not an action is already in the infants’ motor repertoire (van Elk et al., 2008; Gerson et al., 2015). In this line, spectral power in the 7–9 Hz frequency band was more suppressed in 14- to 16-month-olds for the observation of crawling compared to walking (van Elk et al., 2008). This effect was highly related to infants’ own crawling experience in that more experienced crawlers showed stronger mu desynchronization. In addition, mu desynchronization was sensitive to bidirectional action-effect associations (of sounds and rattles) in 8-month-olds (Paulus et al., 2012). In sum, this branch of research indicates that motor activation measured by mu desynchronization depends on experience with stronger reduction of mu frequency power occurring for more familiar or trained actions.

In addition, mu desynchronization can be related to generating action predictions (Stapel et al., 2010; Saby et al., 2012). Stapel et al. (2010) found stronger mu desynchronization on fronto-central and mid-frontal channels in 12-month-olds in response to extraordinary actions (e.g., lifting a cup to the ear) compared to ordinary actions (e.g., lifting a cup to the mouth). The authors interpreted this result by applying the theory of predictive coding (Kilner et al., 2007): According to this theory, the MNS forms predictions about another person’s action given an assumed goal. The MNS constantly checks whether the predicted action goal still matches what is being observed. For unusual action outcomes, like putting a cup to the ear, there is a mismatch between prediction and observation. Consequently, a new prediction has to be generated and this results in stronger motor activation (Gardner et al., 2015).

To summarize, analyzing mu frequency band power allows us to investigate infants’ action processing. While studies on infants’ own action experiences reported increased motor activation when observing more familiar actions, studies manipulating action outcomes found that unexpected outcomes elicit a stronger mu desynchronization than expected outcomes. Thus, the mu frequency seems to be involved in both motor resonance depending on action experiences and on action prediction. However, previous research predominantly investigated mu frequency power in response to actions with familiar objects (e.g., a cup or food). This offers us a unique opportunity to study the cognitive processes during infants’ observation of head-touch actions with novel objects as used in previous behavioral studies on selective imitation. In particular, reduced mu power during unusual head-touch actions (compared to hand-touch actions) would speak for the induction of a prediction error while watching these actions in the absence of situational constraints. On the other hand, stronger mu suppression in response to hand actions would argue for the role of previous motor experience in processing these actions, since infants much more frequently manipulate objects with their hands.

Thus, this is the first study investigating the neural mechanisms underlying the observation of an unusual head touch in adaptation to paradigms previously used in imitation studies (Gergely et al., 2002; Zmyj et al., 2009). Here, we explored possible neuronal mechanisms that might have influenced selective imitation demonstrated in previous studies. In addition, we aimed to elucidate whether these neural mechanisms are sensitive to the action context or not (cf. Zmyj et al., 2009). To examine infants’ neural processing, we designed an EEG study measuring context-dependent motor system activity via mu frequency power during infants’ perception of different action outcomes. In a between-subjects design, 12- to 14-month-olds watched short video sequences of models demonstrating that their hands were free (hands-free condition) or restrained (hands-restrained condition). Subsequent test frames showed the same model turning on a lamp or soundbox using either their head or their hand. We intended to explore whether there were differences in mu power between processing of head- and hand-action outcomes in the hands-free condition and whether mu power varied depending on situational constraints in the hands-restrained condition.

We hypothesized that if prediction error and updating (cf Kilner et al., 2007; Stapel et al., 2010) take place when infants observe others using their head rather than their hand to manipulate an object, then reduced mu power on central channels in response to head actions compared to hand actions should occur in the hands-free condition. In the hands-restrained condition, we expected the opposite result pattern if infants incorporate situational factors while predicting and evaluating action outcomes (i.e., reduced mu power in response to hand compared to head actions). If motor experience influences mu frequency power (van Elk et al., 2008; Gerson et al., 2015), then lower mu power indicating motor resonance in response to familiar hand actions compared to less familiar head actions should be demonstrated in the hands-free condition and possibly also in the hands-restrained condition. If infants do not take into account context information, then results should be similar in both the hands-free and the hands-restrained condition.

## Materials and Methods

### Participants

The final sample consisted of 22 12- to 14-month-old infants (11 girls, *M* = 13 months 2 days, *SD* = 23 days, age range = 12 months 5 days – 14 months 24 days) in the hands-free condition and 20 infants (9 girls, *M* = 12 months 25 days, *SD* = 22 days, age range = 12 months 1 day – 14 months 29 days) in the hands-restrained condition. Infants were recruited from a midsized German city and surrounding areas. They were from middle-class background, born full-term (37–41 weeks of gestation), Caucasian and without any known neurological problems. In addition, 32 infants were tested but excluded from the final sample due to fussiness (i.e., infants showed too many movement artifacts or started crying before being presented with the required number of trials), another 39 infants failed to provide 10 artifact-free trials per within-subjects condition, in 4 additional infants contact of the reference electrode was not satisfactory (i.e., very spiky signal of all electrode channels) and in two sessions technical and experimental errors occurred. This attrition rate is within the typical range for infant EEG studies of 50–75% (e.g., DeBoer et al., 2007; Stets et al., 2012). The loss of participants mainly resulted from 12- to 14-month-olds’ difficulty to sit motionless during the presentation of multiple trials, as it is required for acquiring valid EEG data. There is no indication for a systematic distortion of our sample. Informed verbal and written consent were obtained from each participant’s parent before conducting the experiment. Infants received a certificate with their photo for participation. Experimental procedures were approved by the ethics committee of Friedrich Schiller University in Jena (reference 3752-04/13).

### Stimuli

Infants were presented with video clips and photographs showing adult models performing head or hand actions (adapted from Gergely et al., 2002; Zmyj et al., 2009). Two different types of videos were used: To establish context and motivation at the beginning of the experiment, infants watched two pre-demonstration videos showing a female or male adult sitting at an empty table demonstrating that the hands were free or restrained by turning them. Each participant watched both videos in randomized order regarding sex of the model and situational constraints.

**FIGURE 1:**
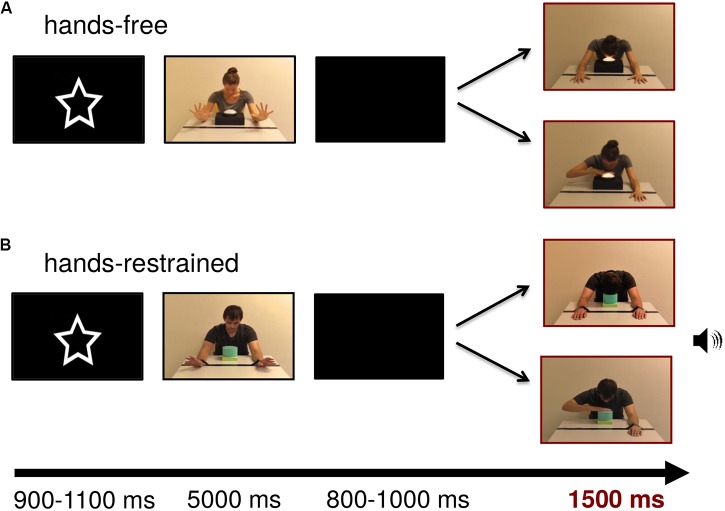
Stimulus examples of lamp and sound modality for **(A)** hands-free and **(B)** hands-restrained condition. Persons appearing in this figure consented to the publication of these images.

Following the pre-demonstration videos, each trial of the demonstration-phase videos illustrated the action context depicting one of four models (two males, two females) sitting at a table with a touch light in front of them. Subsequent test frames depicted action outcomes. In the hands-restrained condition (adapted from Zmyj et al., 2009), the model’s hands were tied to the table with duct tape and could not be moved freely. In the hands-free condition, a line of duct tape was visible on the table but the model’s hands were free. In both conditions, subsequent test frames showed a model turning on a lamp using either their hand or their head (see **Figure 1**). The model did not establish eye contact with the observer during the whole experiment. In half of the trials, a round lamp (12 cm diameter) mounted on a black box (27 cm × 20 cm × 6 cm) was illuminated while the model was touching it (cf Meltzoff, 1988). To increase infants’ attention toward the presentation, in the other half of trials a toy-squeezing sound was generated while the model was touching a blue and green soundbox (13 cm × 13 cm × 11.5 cm) (in accordance with Buttelmann et al., 2007). The sound was presented with a maximum intensity of 75 dB.

In the test frames, the model was presented on screen with a width of approximately 9.13 cm (visual angle of 9.49°) and a height of 10.34 cm (visual angle of 10.74°) measured from head to table. The touch light was presented with a size of 4.6 cm × 8.4 cm (visual angle of 4.79° × 8.73°) and the soundbox with a size of 4 cm × 4.5 cm (visual angle of 4.17° × 4.69°). Test frames were adjusted to each other with Adobe Photoshop CS4 extended in terms of brightness and contrast (all *p*s > 0.05). **Figure 1** depicts example trials in which the model turns on the light or produces a sound by using either his or her hand or head in both conditions.

### Procedure

Infants were tested individually in a quiet, dimly lit room. The testing area was separated from the rest of the laboratory by dark blue colored room dividers. Infants sat on their parent’s lap in front of a 75 Hz 19-inch stimulus monitor with a viewing distance of 55 cm. Parents were instructed not to interact with the infant during data collection. In both the hands-free and hands-restrained condition, the experiment consisted of one block of a maximum of 120 trials. This block comprised 60 trials illustrating a hand touch and 60 trials illustrating a head touch. The videos were displayed in semi-randomized order via the software Presentation (Neurobehavioral Systems, Albany, CA, United States) with the constraint that the same modality (light/sound), gender (male/female) or outcome (hand touch/head touch) were not presented three times consecutively and that all 16 possible test pictures (light/sound, head touch/hand touch, for each of the four models) were displayed within the first 48 trials. To avoid confounding effects of the first observed action, action outcomes (head and hand touch) were counterbalanced between participants in the first trial of each condition.

**Figure 1** shows an exemplary stimulus trial sequence. At the beginning of the trial, a central attractor was presented for an average of 1000 ms to catch infants’ attention. The subsequent video sequence depicted the model showing that the hands were free or restrained by wiggling for 5000 ms. After that, a blank screen was presented for a random period between 800 and 1000 ms. Lastly, the test frame representing hand- or head-action outcomes was presented for 1500 ms. Each trial lasted 8500 ms leading to a maximum total testing time of 17 min. Short breaks could be taken after the end of a trial, when the infant became tired or fussy. The session ended when the infant no longer attended to the screen. EEG activity was recorded continuously and infants were video-recorded throughout the experiment for offline coding of looking behavior and movements.

### EEG Recording and Analyses

Electroencephalogram was measured by a 32-channels ActiCap system (Brain Products, Gilching, Germany) with 32 active silver/silver chloride (Ag/AgCl) electrodes arranged according to the 10–10 system. Horizontal and vertical electrooculograms were recorded bipolarly. Impedances were controlled at the beginning of the experiment and accepted when below 20 kΩ. Sampling rate was set at 250 Hz. Electrode signals were referenced to the right mastoid electrode and amplified via a BrainAmp amplifier.

#### EEG Preprocessing

Electroencephalogram data were first processed by using BrainVision Analyzer 2 (Brain Products, Gilching, Germany) and further analyzed in Fieldtrip (Oostenveld et al., 2011). Raw data were filtered off-line with a 0.3–30 Hz band-pass filter to remove frequencies not related to cortical processes of interest. Data were then re-referenced to the average mastoids (TP9, TP10). Data were automatically excluded if the amplitude of the analyzed channels exceeded a voltage threshold of 200 μV within a 200 ms interval. Thus, data including gross motor movements were rejected from final analysis by this automatic artifact rejection algorithm. Data were then segmented into epochs of waveforms that comprised 200 ms before stimulus onset of the test frame, demonstrating a head touch or a hand touch, through 1500 ms following stimulus onset. Infants’ looking behavior was video-coded offline. Only trials in which infants did not blink and paid attention to the whole presentation of the test frame, showing head- and hand-action outcomes, were included in further analyses. In addition, videos were coded for more subtle movements of infants, such as hand or head movements that resembled actions performed by the video models in our stimuli (i.e., pressing a button by hand or by head or similar actions, like reaching or pointing) (cf Marshall et al., 2011). An independent rater, blind to hypotheses, coded infants’ movements during all observed action outcomes. An additional coder rated 25% of the videos from each condition (hands-free and hands-restrained). A high degree of inter-rater reliability was found between 758 measurements with an average measure intraclass correlation (ICC) of 0.840. To ensure that motor activation related to the target actions (head touch and hand touch) was equivalent between conditions (hands-free and hands-restrained) and within conditions, we conducted a mixed analysis of variance (ANOVA) with the between-subjects factor *condition* (hands-free, hands-restrained) and the within-subjects factor *outcome* (target action movement during head-touch outcomes, target action movement during hand-touch outcomes). The ANOVA did not yield a significant main effect of *outcome*, *F*(1,40) = 2.394, *p* = 0.130, or *condition*, *F*(1,40) = 0.985, *p* = 0.327. Likewise, no significant interaction between *condition* and *outcome* was found, *F*(1,40) = 2.394, *p* = 0.130. Overall, infants very rarely performed actions similar to the hand and head touch demonstrated by the video models during the whole experiment (*M* = 1.69 movements, *SD* = 1.62 movements). Thus, significant differences between conditions and/or action outcomes cannot result from differences in infants’ movements similar to the presented target actions (hand and head touch). Data were then baseline-corrected using 200 ms prior to the onset of the test frame and finally segmented for hand and head touch in both hands-free and hands-restrained conditions, respectively.

#### Frequency Domain Analysis

Artifact-free data segments were submitted to fast Fourier transformations (FFTs). For each segmented test frame (hand or head touch), the power was computed from 0 to 1,500 ms relative to the onset of the related stimulus using a Hanning-tapered window of the same length (by applying the ‘ft freqanalysis’ function with ‘mtmfft’ method as implemented in Fieldtrip). Power estimates were calculated for frequencies (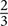 Hz bins) between 0 and 124.667 Hz. Grand averages of the FFTs were computed for both hand- and head-action outcomes in the hands-free and hand-restrained condition.

A minimum of 10 artifact-free trials per outcome was required for an infant to be included in the statistical analyses. In the hands-free condition, each infant contributed 13 to 56 trials (*M* = 21.23, *SD* = 9.88) to the head outcome and 11 to 56 trials (*M* = 19.18, *SD* = 9.81) to the hand outcome. In the hands-restrained condition, each infant contributed 10 to 34 trials (*M* = 17.25, *SD* = 5.87) to the head outcome and 10 to 29 trials (*M* = 16.05, *SD* = 5.45) to the hand outcome. Across conditions each infant contributed 10 to 56 (*M* = 19.33, *SD* = 8.36) valid trials to the head outcome and 10 to 56 valid trials to the hand (*M* = 17.69, *SD* = 8.10) outcome.

**FIGURE 2:**
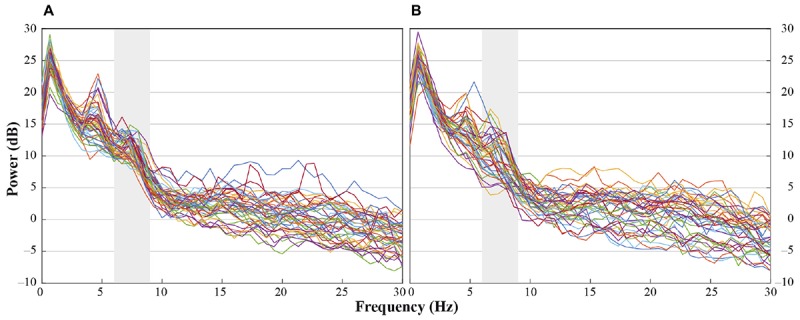
Individual power spectra across an average for hand- and head-touch actions across an average of frontal and central electrodes (F3, F4, C3, C4) for **(A)** hands-free and **(B)** hands-restrained condition.

In accordance with previous research we analyzed central electrode positions on the left and right hemisphere (C3, C4) to investigate differences in motor activation indicated by mu frequency power (e.g., Paulus et al., 2012). As visual inspection indicated differences between unusual head-touch and familiar hand-touch actions especially on frontal channels and as previous studies also investigated the role of frontal activation in infants’ action perception (e.g., van Elk et al., 2008; Stapel et al., 2010), we included lateral frontal channels (F3, F4) into the final analysis. In addition, parietal channels P3 and P4 were included in the analysis in order to exclude the possibility that potential alpha-band effects were widespread across the scalp (including posterior regions) suggesting general arousal rather than involvement of the motor system. Occipital channels (O1, O2) were not selected for comparison to fronto-central electrode positions because channels were too noisy and did not provide enough artifact-free data for valid analyses. For each participant, a dominant mu peak was identified for frontal and central electrodes (F3, F4, C3, C4) between 6 and 9 Hz. Analyses revealed that in the hands-free condition up to 20 infants peaked between 6.7 and 8 Hz in response to the hand touch and up to 19 infants in response to the head touch (see **Figure 2A**). Similarly, in the hands-restrained condition up to 15 infants peaked in response to the hand touch and 15 infants in response to the head touch between 6.7 and 8 Hz (see **Figure 2B**). This is in accordance with previous research on mu frequency in infants indicating that mu frequency band falls between 6 to 9 Hz in infants (Marshall and Meltzoff, 2011) and peaks at about 8 Hz in 1-year-olds (Marshall et al., 2002). Thus, the statistical analyses were conducted across the average power of the 6 to 8 Hz frequency range.

#### Statistical Analysis

To investigate overall differences between conditions, data were analyzed by a mixed ANOVA with the between-subjects factor *condition* (hands-free, hands-restrained) and the within-subjects factors *action outcome* (head, hand), *region of interest* (frontal: F3/F4, central: C3/C4, parietal: P3/P4) and *hemisphere* (left, right). Partial eta squared ($\eta_{p}^{2}$) or Cohen’s *d* (*d*) are reported as estimates of the effect size. Greenhouse-Geisser correction for non-sphericity was employed if applicable for conservative corrections. Fractional degrees of freedom (*df*) were reported when Greenhouse-Geisser correction was necessary (i.e., when Mauchly’s test for sphericity was significant) and applied. The significance level was set at *p* < 0.05 (two-tailed) for all statistical analyses.

## Results

### Hands-Free vs. Hands-Restrained Condition

**FIGURE 3:**
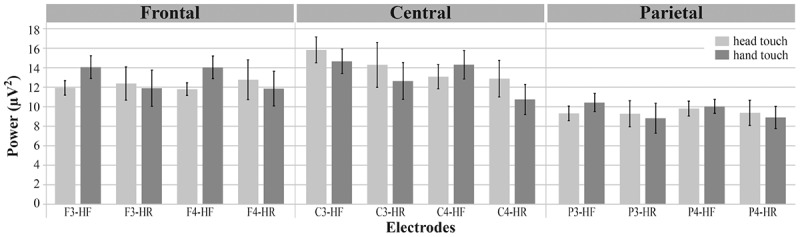
Grand average EEG power across mu frequency band (6–8 Hz) for electrodes of interest (F3, F4, C3, C4, P3, P4) in response to hand touch (dark gray) and head touch (light gray) for both hands-free (HF) and hands-restrained (HF) condition. Error bars represent standard errors of the mean.

To compare results between the hands-free and hands-restrained condition, we first computed a mixed ANOVA with the between-subjects factor *condition* (hands-free, hands-restrained) and the within-subjects factors *action outcome* (head, hand), *region of interest* (frontal, central, and parietal) and *hemisphere* (left, right). Analysis yielded a significant interaction between *condition*, *action outcome*, *region of interest* and *hemisphere*, *F*(2,80) = 3.390, *p* = 0.039, $\eta_{p}^{2}$ = 0.08 (for a detailed illustration of main effects and interactions, see Supplementary Table 1). Thus, conditions were further analyzed separately to explain this interaction effect. Mu power of all electrodes of interest (F3, F4, C3, C4, P3, P4) is plotted in **Figure 3**.

### Hands-Free Condition

**FIGURE 4:**
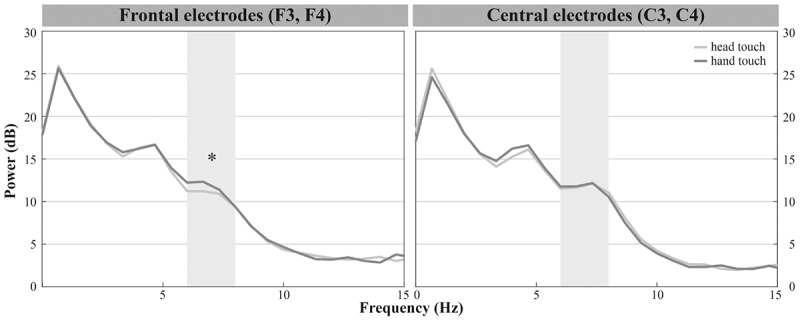
Grand average EEG mu power for hand touch (dark gray) and head touch (light gray) for an average of frontal electrodes (F3, F4) and for an average of central electrodes (C3, C4) in the hands-free condition. Asterisks depict significant differences with *p* < 0.05.

Infants demonstrated dominant peaks in response to observing head- and hand-action outcomes in the frequencies of interest (6–8 Hz) especially on frontal and central electrodes (see **Figure 2A**). Visual inspection of the grand average FFTs indicated reduced mu power in response to the head touch compared to the hand touch. This tendency was more pronounced on frontal electrodes (see **Figure 4**).

The repeated-measures ANOVA (rmANOVA) revealed a significant interaction of *action outcome*, *region of interest* and *hemisphere*, *F*(2,42) = 6.918, *p* = 0.003, $\eta_{p}^{2}$ = 0.25 (for a detailed illustration of main effects and interactions, see Supplementary Table 2). In order to resolve this significant interaction, we conducted three two-way rmANOVAs with the within-subjects factors *action outcome* (head, hand) and *hemisphere* (left, right) for each region of interest. For frontal channels (F3, F4), we found a significant main effect of *action outcome*, *F*(1,21) = 8.675, *p* = 0.008, $\eta_{p}^{2}$ = 0.29, indicating that mu power in both frontal electrodes was significantly lower in response to head-touch outcomes (*M* = 11.87, *SD* = 2.96) compared to hand-touch outcomes (*M* = 14.05, *SD* = 5.35) independent of *hemisphere*, *F*(1,21) = 0.28, *p* = 0.868. Analysis of frontal regions did not reveal a significant interaction between *action outcome* and *hemisphere*, *F*(1,21) = 0.044, *p* = 0.836. For central channels (C3, C4), the rmANOVA analysis yielded a significant interaction of *action outcome* and *hemisphere*, *F*(1,21) = 7.990, *p* = 0.010, $\eta_{p}^{2}$ = 0.28. *Post hoc* *t*-tests for left (C3) and right (C4) hemisphere compared mu frequency power of hand- and head-action outcomes. On the right hemisphere mu power was slightly lower in response to head-touch (*M* = 13.09, *SD* = 5.86) compared to hand-touch outcomes (*M* = 14.31, *SD* = 6.89). However, it did not reach significance, *t*(21) = -1.932, *p* = 0.067, *d* = 0.41. No significant differences in mu power were found on the left hemisphere, *t*(21) = 1.175, *p* = 0.253. For parietal channels (P3, P4), the rmANOVA did not reveal a significant main effect of *action outcome*, *F*(1,21) = 1.076, *p* = 0.311, *hemisphere*, *F*(1,21) = 0.004, *p* = 0.952, nor a significant interaction between *action outcome* and *hemisphere*, *F*(1,21) = 1.869, *p* = 0.186.

Thus, we found reduced mu power in response to head-touch actions compared to hand-touch actions especially on frontal electrode positions and a tendency for the same effect at the right central electrode site.

### Hands-Restrained Condition

**FIGURE 5:**
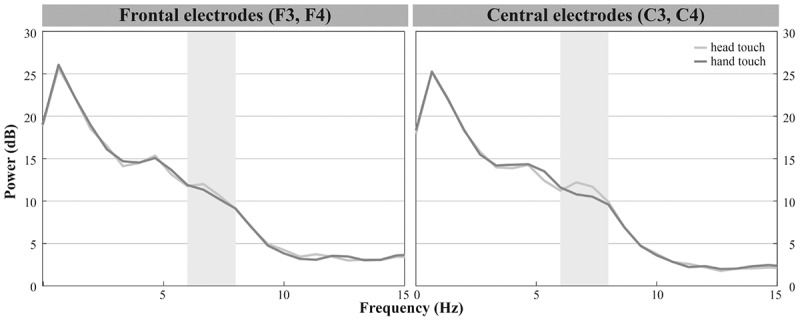
Grand average EEG mu power for hand touch (dark gray) and head touch (light gray) for an average of frontal electrodes (F3, F4) and for an average of central electrodes (C3, C4) in the hands-restrained condition.

In the hands-restrained condition we investigated whether infants incorporate contextual information while evaluating action outcomes via the motor system measured by differences in mu frequency power. Comparable to the hands-free condition, the majority of infants peaked in response to observing head- and hand-action outcomes in the frequencies of interests (6 – 8 Hz) especially on frontal and central electrodes (see **Figure 2B**). Visual inspection indicated increased mu power in response to the head touch and reduced mu power in response to the hand touch (see **Figure 5**).

We again conducted a rmANOVA with the within-subjects factors *action* *outcome* (head, hand), *region of interest* (frontal, central, parietal) and *hemisphere* (left, right). There were, however, no significant interactions of *action outcome*, *region of interest*, and *hemisphere*, all *p*s > 0.29. Likewise the analyses did not reveal main effects of *action outcome*, *F*(1,19) = 1.601, *p* = 0.221 or *hemisphere*, *F*(1,19) = 0.753, *p* = 0.396. We only found a significant main effect of *region of interest*, *F*(2,27.25) = 15.220, *p* < 0.001, $\eta_{p}^{2}$ = 0.45, indicating that across action outcomes overall mu power was lower at parietal regions (*M* = 9.09, *SD* = 5.49) than at frontal (*M* = 12.23, *SD* = 7.60) and central regions (*M* = 12.64, *SD* = 7.79). In sum, results showed no differences in mu power between head and hand touch in the hands-restrained condition.

## Discussion

This study was designed to shed light on the neural mechanisms underlying infants’ observation of unusual head-touch actions used previously in selective imitation studies (e.g., Gergely et al., 2002; Gellén and Buttelmann, 2017). For this, we investigated the role of motor activation through measuring differences in mu frequency power. In addition, we aimed to explore whether motor activation during action perception is sensitive to contextual factors. To this end, we adapted a well-known behavioral imitation paradigm (Gergely et al., 2002; Zmyj et al., 2009) to an EEG experiment for the first time: In a between-subjects design, infants were presented with short video sequences of adult models demonstrating that his or her hands were either free or restrained. Subsequent test frames showed the same person turning on a lamp or soundbox using their head or their hand. Results in the hands-free condition revealed that 12- to 14-month-old infants displayed reduced mu frequency power in response to unusual head-touch actions compared to familiar hand-touch actions. Interestingly, in the hands-restrained condition we did not find differences in mu frequency power in response to hand- vs. head-touch actions.

Previous research associated mu desynchronization with motor activation or cortical processing of movement-related activity (Fox et al., 2016). In the hands-free condition, significant changes in mu frequency band in response to the observation of head-touch vs. hand-touch actions were predominantly found in frontal regions (F3, F4). Despite other studies demonstrating changes in mu frequency band on frontal or fronto-central channels (van Elk et al., 2008; Stapel et al., 2010), effects of mu frequency band are often more pronounced on central electrode positions (Marshall and Meltzoff, 2011). Since in our study no significant differences in mu power were found on central channels, an interpretation of our results in terms of alpha power associated with general attention or cognitive control processes unrelated to motor activation may be considered (Marshall et al., 2009; Quandt et al., 2011; Klimesch, 2012).

In adults, tasks-related modulations in alpha power can be associated with two controlled functions of attention, namely selection and suppression. Here, alpha frequency activity is thought to function as an attention filter and a decrease in alpha amplitude reflects a release from inhibition. In addition, alpha-band activity has been suggested to indicate controlled access of a semantic knowledge system (Klimesch, 2012). Alpha desynchronization across the whole scalp was reported in 9-month-old infants in response to objects that were presented after engaging in mutual eye contact vs. no eye contact. Eye contact might have put infants in a receptive state of semantic knowledge acquisition (Hoehl et al., 2014). According to these accounts, infants may have been more attentive in response to the unusual head touch.

However, we found significant differences in alpha power between unusual and familiar actions only on frontal sites (parietal channels did not show the same result pattern). This is in line with previous neurophysiological studies relating changes in frontal alpha rhythm to early states of observational and imitative learning (Marshall et al., 2009; Quandt et al., 2011). Accordingly, brief imitative experience of unfamiliar actions is associated with larger alpha desynchronization on frontal channels (Marshall et al., 2009) independent of the type of training (visual and/or active experience; Quandt et al., 2011). Thus, neural processing of action observation, especially on frontal channels, is influenced by a moderate amount of initial experience with these actions. Neuroimaging literature suggests that this frontal activation for unfamiliar actions reflects dorsolateral prefrontal cortex (DLPFC) activation during an active process of consolidating or forming motor representations of previously unknown actions (Jeannerod, 2006; Vogt et al., 2007). With increasing active experience, activation shifts toward more posterior motor regions for high levels of expertise (Shadmehr and Holcomb, 1997; Calvo-Merino et al., 2005; Kelly and Garavan, 2005). In this view, the reduction in alpha power on frontal channels in response to unusual head-touch actions compared to hand-touch actions may reflect a process of mapping observed movements onto previously created motor memories (Jeannerod, 2006; Marshall et al., 2009).

Finally, we suggest a third explanation for the frontal effects in the hands-free condition based on our hypotheses. If prediction error and updating (Kilner et al., 2007) take place when infants observed an unusual action, we expected reduced mu power in response to unusual compared to familiar action outcomes (Stapel et al., 2010). If motor experience influenced mu frequency power in the present study, lower mu power in response to familiar hand actions compared to unfamiliar head actions was expected (van Elk et al., 2008; Gerson et al., 2015). We found reduced mu power in response to the unusual head touch compared to the familiar hand touch and, thus, propose that infants updated their action predictions via the motor system for action outcomes that violated their prior action expectations (Kilner et al., 2007).

Our neural findings are in line with previous behavioral research on action understanding suggesting that by the age of 6 months infants are able to predict another person’s actions (for a similar explanation of the results, see principle of rationality, Gergely and Csibra, 2003). For example, 6-month-olds anticipated action outcomes more frequently for functional compared to non-functional goal-object combinations (e.g., cup to mouth or to ear) or their pupils dilated in response to unexpected feeding actions (Gredebäck and Melinder, 2010; Hunnius and Bekkering, 2010). In addition, our results are in accordance with previous EEG studies on action processing. In the hands-free condition, we replicated the finding by Stapel et al. (2010) that 12-month-olds showed stronger mu desynchronization in response to extraordinary compared to ordinary actions. Further EEG studies demonstrated that even 9-month-old infants discriminated familiar vs. unusual eating actions. Infants responded with an N400-like component only to unexpected action outcomes (e.g., pretzel put to ear) indicating a violation of semantic action context (Reid et al., 2009; Kaduk et al., 2016). Furthermore, infants have been shown to distinguish between disrupted and complete actions in terms of increased frontal gamma band activity or more negative slow wave components (Reid et al., 2007; Pace et al., 2013). However, low-level explanations (e.g., variability in stimulus materials) might have accounted for differences between conditions in previous studies. To sum up, in the hands-free condition reduced mu power in response to the unusual head touch indicates that 12- to 14-month-old infants were able to predict action outcomes after being presented with the action context.

In addition, we investigated whether context information influenced motor activation in the hands-restrained condition. We expected opposite result patterns to the hands-free condition. Accordingly, the head touch did not elicit lower mu power compared to hand touch in the hands-restrained condition. Thus, it seems that infants incorporate situational factors while evaluating action outcomes. This is in accordance with previous behavioral studies suggesting that by 6–12 months of age infants are able to interpret actions as goal-directed and take into account situational constraints (e.g., Gergely et al., 1995; Woodward and Sommerville, 2000; Schwier et al., 2006; Zmyj et al., 2009; Gredebäck and Melinder, 2010). Despite visual inspection indicating differences in mu power especially on central channels, we did not find significant different brain responses between hand- and head-action outcomes in the hands-restrained condition. In line with previous behavioral and imitation studies (Gergely and Csibra, 2003; Schwier et al., 2006; Zmyj et al., 2009), we would have expected infants to discriminate both action outcomes also in this scenario. The predictive coding theory proposes that the MNS functions to recognize and code for goals of observed actions (Kilner et al., 2004, 2007). Infants should have been able to encode both action goals and context-specific information to predict action outcomes and update their predictions in case of prediction error. When observing a model turning on a lamp by hand despite the fact that hands were previously tied to the table, prediction error and prediction updating were expected to take place in response to the physically impossible action.

There are several possible explanations for why we did not find differences between hand and head touches in the hands-restrained condition. First, infants might have not entirely processed the restraining duct tape visually. Second, it might be that infants did not know what to predict when they observed a person whose hands were tied to the table. In this case subsequent action outcomes would have not been evaluated in comparison to prior action predictions (for a similar explanation in word learning by exclusion, see Grassmann et al., 2015). These explanations are rather unlikely, as Zmyj et al. (2009) demonstrated that 1-year-olds imitated selectively depending on the same external physical constraint when presented on a computer screen. Besides, if infants did not recognize our situational constraint at all, results should have revealed similar effects to the hands-free condition. Another explanation might be that infants visually processed the situational constraint but the head touch was still highly salient. This hypothesis is supported by a recent eye-tracking study demonstrating that 14-month-old infants paid a similar high amount of attention to the head touch of a model irrespective of whether or not the model was able to use his or her hands (Buttelmann et al., 2017).

Finally, two different processes might have played a role in the hands-restrained condition: One-year-olds already have numerous experiences with hand-touch actions as they can observe other humans turning on switches resulting in visual (e.g., light) or auditory effects (e.g., sounds) repeatedly in everyday life. Increased experience might have enhanced motor activation at central sites during action observation (van Elk et al., 2008; Cannon et al., 2014; Gerson et al., 2015). In addition, infants might have formed action predictions based on semantic knowledge. Action outcomes that violated these prior predictions might have led to prediction updating and, thus, increased motor activation (Kilner et al., 2007). Both high experience and prediction updating in response to hand actions might have affected mu power at the same time in the hands-restrained condition. Hence, we conclude that motor activation measured via mu frequency band is context-sensitive in the present study. However, effects of experience might have interfered with brain activity based on predictive coding. This is in accordance with an adult study measuring influences of motor experience and conceptual knowledge on brain activity in action perception (Gerson et al., 2017). Here, motor experience and predictions based on conceptual familiarity were experimentally manipulated in a 1-week pre-/post-training design. Results revealed that motor system activity measured via beta power changed in response to both factors in a parallel but distinct way: Increased experience led to increased motor activity whereas increased conceptual information about a previously unfamiliar action led to a relative decrease of motor activity across time. To summarize, results of the hands-restrained condition differed from the hands-free condition in terms of mu power indicating that mu power reflecting motor activation during action observation is context-dependent.

The stimuli used in the present study were based on previous behavioral imitation studies indicating that 12- to 14-month-olds are more likely to imitate an unusual head touch depending on varying situational constraints (Gergely et al., 2002; Zmyj et al., 2009). Our neural findings extend recent behavioral results as we revealed differences in mu power in response to head vs. hand touch dependent on external situational constraints. In addition, our results suggest a neural mechanism underlying previous behavioral findings: Infants might form action predictions and update their predictions for deviating action outcomes via the motor system (Kilner et al., 2007). In accordance with the predictive coding framework, increased motor activation in response to the unusual head touch might reflect the process of updating predictions in case of prediction error. This is in line with research on adults demonstrating increased motor activation in response to deviating or unusual action outcomes (e.g., Manthey et al., 2003; Koelewijn et al., 2008). Motor system activity in adults was even sensitive to the degree of prediction with increased activation in response to highly predictable action outcomes (Braukmann et al., 2017).

The present results highlight the role of motor activation during action perception by utilizing stimuli adapted to previous behavioral studies. However, with the present neurophysiological findings we cannot draw any conclusions regarding the possible effect on infants’ imitative behavior. Here, we offer one possible explanation for why infants show increased motor activation in response to unusual actions; this explanation is in accordance with the predictive coding theory. The relation between motor activation and infants’ imitation still awaits further clarification.

In sum, the present study revealed a reduction in mu power, which might be related to the motor system, in response to an unusual head-touch action in 12- to 14-month-old infants. Reduced mu power in response to unusual compared to familiar actions may indicate prediction error and updating according to the predictive coding framework (Kilner et al., 2007). This effect was only pronounced in the hands-free condition, suggesting that the motor system activated during action prediction and evaluation is context-dependent. Our neuroscientific findings extend previous behavioral results suggesting that a reduction of mu frequency power is one possible functional mechanism underlying infants’ early action understanding.

## Ethics Statement

This study was conducted in the Baby Laboratory of the Department of Biological and Developmental Psychology at Heidelberg University, Heidelberg, Germany. The study and experimental procedures were approved by the ethics committee of Friedrich Schiller University, Jena, Germany (reference: 3752-04/13) and were in accordance with the Declaration of Helsinki. Participants were recruited from a database of parents interested in participating in infant studies at the Department of Biological and Developmental Psychology at Heidelberg University, Heidelberg, Germany. Parents of all subjects gave written and verbal consent before conducting the experiment.

## Author Contributions

ML, DB, SG, SP, and SH conceived and designed the study. ML collected the data. ML and DM analyzed the data. SH was consulted about data interpretation. ML drafted the manuscript. All authors revised the work and approved the final version for publication.

## Conflict of Interest Statement

The authors declare that the research was conducted in the absence of any commercial or financial relationships that could be construed as a potential conflict of interest.
