# Corrigendum: Reduced Mu Power in Response to Unusual Actions Is Context-Dependent in 1-Year-Olds

**DOI:** 10.3389/fpsyg.2019.00316

**Published:** 2019-02-20

**Authors:** Miriam Langeloh, David Buttelmann, Daniel Matthes, Susanne Grassmann, Sabina Pauen, Stefanie Hoehl

**Affiliations:** ^1^Max Planck Institute for Human Cognitive and Brain Sciences, Leipzig, Germany; ^2^Department of Psychology, Heidelberg University, Heidelberg, Germany; ^3^Department of Psychology, University of Bern, Bern, Switzerland; ^4^Institute of Educational Research and Development, University of Applied Sciences and Arts Northwestern Switzerland, Windisch, Switzerland; ^5^Faculty of Psychology, University of Vienna, Vienna, Austria

**Keywords:** EEG, infants, action perception, action understanding, mu frequency, mirror neuron system

In the original article, there were mistakes in Figures 2–5 as published. We analyzed the artifact-free data segments in Fieldtrip (Oostenveld et al., 2011) using the “ft_freqanalysis” function. We configured this function to compute power, however, stated erroneously in the original text that we computed the “power spectral density (PSD).” Consequently, we labeled the y-axis units according to PSD but not power.

**Figure 2:**
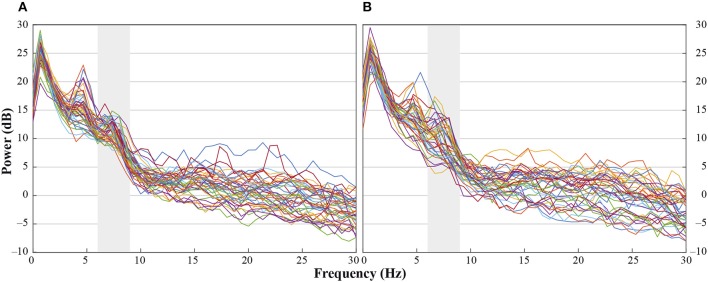
Individual power spectra across an average for hand- and head-touch actions across an average of frontal and central electrodes (F3, F4, C3, C4) for **(A)** hands-free and **(B)** hands-restrained condition.

The y-axis unit in Figure 3 was corrected to “μV^2^”, additionally, the scaling used in Figures 2, 4 and 5 was a natural logarithm instead of a common logarithm. The scaling has now been adjusted to the common logarithm and the y-axis unit has been adjusted to “dB” accordingly. The corrected Figures 2–5 appear below.

**Figure 3:**
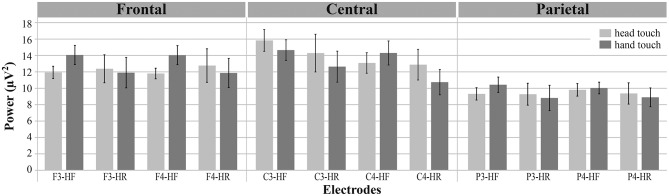
Grand average EEG power across mu frequency band (6–8Hz) for electrodes of interest (F3, F4, C3, C4, P3, P4) in response to hand touch (dark gray) and head touch (light gray) for both hands-free (HF) and hands-restrained (HF) condition. Error bars represent standard errors of the mean.

**Figure 4:**
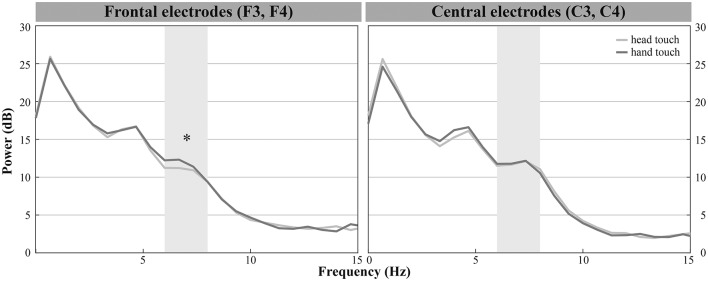
Grand average EEG mu power for hand touch (dark gray) and head touch (light gray) for an average of frontal electrodes (F3, F4) and for an average of central electrodes (C3, C4) in the hands-free condition. Asterisks depict significant differences with *p* < 0.05.

**Figure 5:**
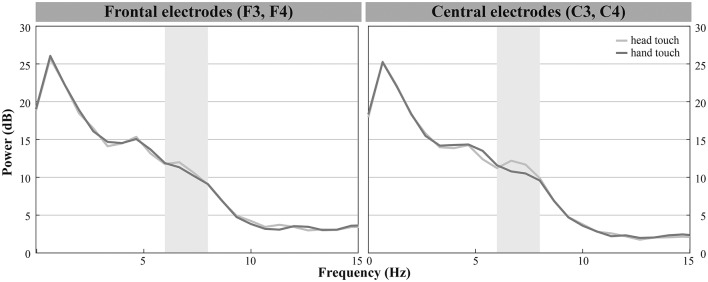
Grand average EEG mu power for hand touch (dark gray) and head touch (light gray) for an average of frontal electrodes (F3, F4) and for an average of central electrodes (C3, C4) in the hands-restrained condition.

A correction has also been made to the ***Materials and Methods, EEG Recording and Analyses, Frequency Domain Analysis, Paragraph one**:*

“Artifact-free data segments were submitted to fast Fourier transformations (FFTs). For each segmented test frame (hand or head touch), the power was computed from 0 to 1,500 ms relative to the onset of the related stimulus using a Hanning-tapered window of the same length (by applying the ‘ft freqanalysis’ function with ‘mtmfft’ method as implemented in Fieldtrip). Power estimates were calculated for frequencies ($\frac{2}{3}$Hz bins) between 0 and 124.667 Hz. Grand averages of the FFTs were computed for both hand- and head-action outcomes in the hands-free and hand-restrained condition.”

Additionally, there was a mistake in the legend for Figure 2 as published. The legend has been rewritten to provide a better understanding of the figure content. The correct legend appears below.

“Figure 2. Individual power spectra across an average for hand- and head-touch actions across an average of frontal and central electrodes (F3, F4, C3, C4) for (A) hands-free and (B) hands-restrained condition.”

The authors apologize for these errors and state that they do not change the scientific conclusions of the article in any way. The original article has been updated.
