# Supplementary material for: Reduced Mu Power in Response to Unusual Actions Is Context-Dependent in 1-Year-Olds
Source: Front Psychol. 2018 Jan 30;9:36. doi: 10.3389/fpsyg.2018.00036 (PMC5797571; doi:10.3389/fpsyg.2018.00036)
Supplement: Supplementary file 1 [file Table_1.DOCX]

Supplementary Material

Reduced mu power in response to unusual actions is context-dependent in 1-year-olds

Miriam Langeloh*, David Buttelmann, Daniel Matthes, Susanne Grassmann, Sabina Pauen, Stefanie Hoehl

*** Correspondence:** Corresponding Author: langeloh@cbs.mpg.de

# Supplementary Tables

Table 1

*2 (condition: hands-free, hands-restrained) x 2 (action outcome: head, hand) x 3 (region of interest: frontal, central, parietal) x 2 (hemisphere: left, right) mixed ANOVA table*

| Source | *df* | *F* | *η_p_^2^* | *p* |
| --- | --- | --- | --- | --- |
| cond ^a^ | 1 | 0.48 | .01 | .493 |
| error (cond) ^a^ | 40 |  |  |  |
| outc | 1 | 0.00 | .00 | .950 |
| outc x cond | 1 | 4.31* | .10 | .044 |
| error (outc) | 40 |  |  |  |
| ROI | 2 | 24.53** | .38 | < .001 |
| ROI x cond | 2 | .52 | .01 | .600 |
| error (ROI) | 80 |  |  |  |
| hemis | 1 | 1.70 | .04 | .200 |
| hemis x cond | 1 | .01 | .00 | .936 |
| error (hemis) | 40 |  |  |  |
| outc x ROI | 2 | 3.55* | .08 | .033 |
| outc x ROI x cond | 2 | .94 | .02 | .395 |
| error (outc x ROI) | 80 |  |  |  |
| outc x hemis | 1 | .12 | .00 | .733 |
| outc x hemis x cond | 1 | 1.55 | .04 | .220 |
| error (outc x hemis) | 40 |  |  |  |
| ROI x hemis | 1.57 | 2.96 | .07 | .071 |
| ROI x hemis x cond | 2 | .03 | .00 | .974 |
| error (ROI x hemis) | 62.89 |  |  |  |
| outc x ROI x hemis | 2 | 2.08 | .05 | .132 |
| outc x ROI x hemis x cond | 2 | 3.39* | .08 | .039 |
| error (outc x ROI x hemis) | 80 |  |  |  |

*Note.* cond = condition; outc = action outcome; ROI = region of interest; hemis = hemisphere.

^a^ = between-subjects factor

* *p* < .05 ** *p* < .001
