# Supplementary material for: Reduced Mu Power in Response to Unusual Actions Is Context-Dependent in 1-Year-Olds
Source: Front Psychol. 2018 Jan 30;9:36. doi: 10.3389/fpsyg.2018.00036 (PMC5797571; doi:10.3389/fpsyg.2018.00036)
Supplement: Supplementary file 2 [file Table_2.DOCX]

Supplementary Material

Reduced mu power in response to unusual actions is context-dependent in 1-year-olds

Miriam Langeloh*, David Buttelmann, Daniel Matthes, Susanne Grassmann, Sabina Pauen, Stefanie Hoehl

*** Correspondence:** Corresponding Author: langeloh@cbs.mpg.de

# Supplementary Tables

Table 2

*2 (action outcome: head, hand) x 3 (region of interest: frontal, central, parietal) x 2 (hemisphere: left, right) repeated-measures ANOVA table*

| Source | *df* | *F* | *η_p_^2^* | *p* |
| --- | --- | --- | --- | --- |
| outc | 1 | 3.17 | .13 | .090 |
| error (outc) | 21 |  |  |  |
| ROI | 2 | 14.18** | .40 | < .001 |
| error (ROI) | 42 |  |  |  |
| hemis | 1 | 0.96 | .04 | .339 |
| error (hemis) | 21 |  |  |  |
| outc x ROI | 2 | 3.99* | .16 | .026 |
| error (outc x ROI) | 42 |  |  |  |
| outc x hemis | 1 | 1.48 | .06 | .238 |
| error (outc x hemis) | 21 |  |  |  |
| ROI x hemis | 2 | 1.56 | .07 | .221 |
| error (ROI x hemis) | 42 |  |  |  |
| outc x ROI x hemis | 2 | 6.92* | .25 | .003 |
| error (outc x ROI x hemis) | 42 |  |  |  |

*Note.* outc = action outcome; ROI = region of interest; hemis = hemisphere.

* *p* < .05 ** *p* < .001
